# Ag Microparticle/Au Nanoparticle Thermal Interface Materials Sintered at Low Temperature and Pressure

**DOI:** 10.3390/ma18214981

**Published:** 2025-10-31

**Authors:** Krzysztof Stojek, Adam Krzysztof Nowak, Olga Rac-Rumijowska, Mateusz Czok, Damian Nowak, Przemysław Matkowski

**Affiliations:** Faculty of Electronics, Photonics and Microsystems, Wrocław University of Science and Technlogy, Wyb. Wyspiańskiego 27, 50-370 Wrocław, Poland; 268577@student.pwr.edu.pl (A.K.N.); olga.rac-rumijowska@pwr.edu.pl (O.R.-R.); mateusz.czok@pwr.edu.pl (M.C.); damian.nowak@pwr.edu.pl (D.N.); przemyslaw.matkowski@pwr.edu.pl (P.M.)

**Keywords:** thermal joint, thermal management, gold, silver, nanoparticles, sintering, electronics packaging

## Abstract

**Highlights:**

**What are the main findings?**

- Silver- and gold-based thermal interface materials (TIMs) are made by low-temperature sintering, where nearly all investigated materials demonstrated a mechanical strength higher than the 6 MPa required by the military standards (MIL-STD-883K);
- A beneficial impact of increased sintering temperature on the mechanical parameters of the sintered joints was observed, but it also resulted in a significant increase in the material’s thermal resistance.

**What are the implications of the main findings?**

- Materials composition requires further research in order to improve thermal performance, where mechanical parameters are satisfactory.

**Abstract:**

This article describes the preparation of thermal interface materials (TIMs) based on silver microparticles and gold nanoparticles, fabricated via low-temperature sintering, and evaluates their mechanical and thermal properties. The characterization involved several techniques, including shear strength testing, metallographic analysis, and thermal resistance measurements. A comparative analysis of the key material properties, namely thermal and mechanical performance, was conducted. The results identify the 2:1 LC formulation—comprising silver microparticles, gold nanoparticles, glycol, and ethanol at a weight ratio of 2:0.01:7:5—as the most effective among the tested TIMs. Furthermore, a positive correlation between increased sintering temperature and enhanced mechanical properties was observed.

## 1. Introduction

Since the invention of the junction transistor and the subsequent development of mass production techniques for these components, an increase in their integration density has been observed, with their count doubling approximately every two years in accordance with Moore’s Law [1]. This trend necessitates ever more efficient heat dissipation, and consequently, the development of thermal interface materials (TIMs) exhibiting high thermal conductivity while maintaining adequate mechanical and electrical performance. To mitigate these issues, various heat dissipation methods are employed, including conduction, convection, and radiation [2]. The relative contribution of each mechanism depends on the ambient conditions and the specific device under consideration [3]. For example, under vacuum conditions, radiation is the only effective mode of heat transfer. Conduction, the most efficient of these mechanisms, entails heat transport through thermally conductive media, such as metals. Two fundamental modes of thermal conduction can be distinguished. Electronic conduction, predominant in metals, arises from the transport of energy by free electrons over relatively long distances [4,5]. The second mechanism is photonic conduction, associated with elastic wave propagation due to lattice vibrations in the metal crystal [6]. The overall thermal conductivity is additive, comprising the sum of these two contributions. Convection relies on the transfer of energy between fluid molecules (either liquid or gas) that are free to move within a given volume. Upon absorption of thermal energy, the heated fluid decreases in density, causing the buoyant rise of warmer regions [7]. The final mechanism, radiation, is comparatively negligible below 100 °C and is based on the emission of electromagnetic waves, requiring no material medium. Radiation can occur only from bodies whose thermal energy exceeds the energy they absorb from their surroundings; thus, such bodies cool by radiative emission [6].

When mating two surfaces, microscale irregularities trap air, thereby impeding heat flow and introducing thermal resistance; surface roughness itself further contributes to this resistance. TIMs are engineered to conform to these irregularities as completely as possible, thus reducing interfacial gaps and enhancing thermal conductivity while preserving high mechanical strength. Many different types of TIMs can be found in the literature [8,9,10], especially for high power electronics applications [11]. These mostly include thermal greases, adhesives, gels [12], phase change materials [13], and graphene-epoxy composites [14]. In [15,16], the authors reported significant improvements in both the mechanical properties and thermal conductivity of sintered interfaces coated with a thin silver layer. To ensure stable and repeatable measurements of thermal resistance, they controlled environmental conditions and minimized convective heat transfer during testing. Among the TIMs evaluated, the best mechanical performance was exhibited by a formulation comprising 95 wt% silver nanoparticles and 5 wt% resin.

The principal aim of producing silver-based TIMs with gold nanoparticles is to mitigate the detrimental effects of oxidation in the silver–silver interface by forming a protective gold layer. Moreover, chemical synthesis of gold nanoparticles permits precise control over particle parameters, enabling the fabrication of nanoparticles with well-defined shapes, size distributions, and surface functionalization [17]. This approach holds promise for achieving more uniform and durable sintered interfaces with enhanced mechanical strength and only marginal increases in thermal resistance. Consequently, such nanoparticles find applications not only in medicine and catalysis [18] but also in electronics, for instance, in modifying sensor layers in gas detectors [19].

Our previous studies focused on the evaluation of thermally conductive materials such as blends of micro- and nano-particles in the form of inks, pastes, and hybrid composites. The samples were subjected to non-destructive analyses of their structure using X-ray computed tomography, as well as comparative thermal analyses of various compositions used in demonstrators (benchmark tests) that simulate real applications. We also evaluated the durability of sintered silver interconnections during accelerated reliability testing (vibrations and vibrations combined with temperature cycles) [20,21].

Based on our experience gained over several years, we are currently focused on applying a mixture of nano- and microparticles of Ag and Au in the sintering process for electronic packaging. We are going to evaluate and optimize the entire process, from material development through material application, characterization, and assessment of the long-term stability and durability of the demonstrators. This article describes first step of the process. Section 2 describes the experimental methods, including sample preparation and methods of thermal, electrical, and structural analysis. Section 3 presents the results and discussion on the materials, including the gold and silver morphology, TIM morphology, and the thermal, mechanical, and comparative analyses. Section 4 presents the conclusions.

## 2. Materials and Methods

### 2.1. Sample Preparation

**Silver deposition**. Two types of silicon substrates were used for sample preparation: thin substrates (4 inches, 300–400 μm thick) and thick (2 inches, 3 mm thick). All substrates were subjected to one-sided texturing and the removal of silicon oxide through etching with concentrated hydrofluoric acid. Then, using magnetron sputtering (Pfeiffer Classic 570, Pfeiffer Vacuum Technology Ag, Asslar, Germany), a thin layer of pure silver (approximately 1 μm) was deposited onto the surface from 99.99% silver target (Table 1). To improve the diffusion of silver, the substrates were annealed in a Snol 8.5/1100 (P) muffle furnace (SnolTherm, UAB, Pabradė, Lithuania) with the following parameters: temperature of 600 °C, heating rate of 10 °C/min, duration of 120 min. The optimal process conditions were determined based on previous studies [22].

**Silicon dicing.** This was followed by precision cutting of wafers into 5 × 5 mm^2^ samples, with two different methods depending on the substrate thickness. Thick substrates were cut with a diamond cutting wheel on a Secotom-50 precision cutting machine (Struers ApS, Ballerup, Denmark), resulting in defect-free chips measuring 5 × 5 × 3 mm^3^. Thin substrates were cut using a UV laser system (LPKF ProtoLaser U, LPKF Laser & Electronics SE, Garbsen, Germany), where multiple cuts resulted in consistent dimensions. Finally, to remove contaminants and degrease the chips, they were treated with isopropyl alcohol using an ultrasonic cleaner. For thick chips, an additional rinsing in boiling deionized water was applied prior to this process to remove the beeswax residues used to fit the substrates into the Secotom-50. This multi-step technological process ensured the production of high-quality test samples with precisely controlled parameters.

**Thermal interface material preparation and deposition.** TIM mixtures were prepared by weighing different amounts of investigated substances. As metal particles, silver microparticles from the Institute of Electronic Materials Technology (Łukasiewicz–ITME, Warsaw, Poland) and gold colloids were used. The gold nanoparticles were synthesized by a chemical reaction using polyvinylpyrrolidone (PVP) as a stabilizing agent. A description of the synthesis can be found in our previous publication [17]. In order to obtain a solution with gold content higher than 200 ppm, the solution was freeze-dried (−47 °C, 2 mPa), which reduced its volume by about 35 times. The final nanoparticle colloid had a gold concentration of 0.69 wt%.

TIM material samples were made in 10 mL glass vessels. The samples were divided into two batches, both of which used the same weight ratios of silver to gold. However, in one batch, twice the amount of solvents was added. The batches were designated as LC and HC based on the low and high concentrations of metal in the mixture. The solid phase mass in the samples was approximately 400 mg, and the weight ratios of the various ingredients varied (Table 2).

Silver microparticles, gold nanoparticles, and measured amounts of solvents were added to a vessel and mechanically stirred until they became homogeneous, then sonified in a VCX-130 (Sonics & Materials, Inc., Newtown, CT, USA) for 2 min at 30 kHz and 50% signal amplitude. Then, the prepared mixtures were immediately applied to the previously prepared silicon substrates.

**Figure 1:**
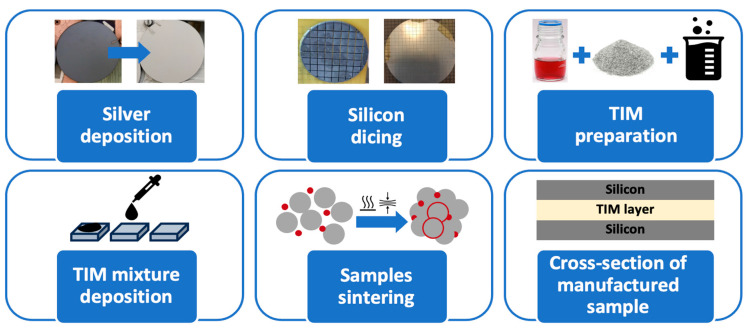
Sample preparation.

The process of preparing, and sintering the samples consisted of several steps (Figure 1). First, the silicon substrates were cleaned in a bath of isopropyl alcohol or acetone. Then, using a glass dipstick, a previously prepared thermal interface mixture was applied to the metallized side of the chip. Once a drop of the mixture was placed on one half of the sample, it was covered with the other half to form a complete test sample. The schematic drawing shows this arrangement.

**Sample sintering.** The samples were placed in a configuration resembling a ‘three-legged chair’, which included three samples on an aluminum plate covered with a thin polyimide film to prevent them from sticking. They were then covered with a second aluminum plate, which was loaded to ensure uniform pressure of approximately 0.5 MPa on each structure, where typical pressures for metal sintering reach approximately 20 to 40 MPa. In industrial electronics packaging, using pressures in the range of tens of megapascals can lead failures of the assembled elements; therefore, reducing the pressure becomes crucial. Conversely, investigations are also being conducted on pressureless die-attach sintering methods. A similar approach is being explored using temperatures. Typical sintering of silver is conducted in temperatures below its melting point (961 °C), i.e., 800 °C. Such a high value is not useable for typical die-attach sintering at the first (especially) or second level of electronics packaging. Substrates and other elements of a given system might be damaged. Lowering the temperature to 230 °C is beneficial for process safety; however, this may affect thermal joint quality. In our research, both pressure and temperature are lower than in typical sintering processes for thermal joint manufacturing. In addition, the temperature and pressure are related and require optimization. Even under low pressure and low-temperature sintering conditions, components may be damaged. Therefore, it is necessary to adjust their values to the required electronic component. If a component is damaged by temperature, the temperature can be reduced and the pressure increased (or vice versa) to produce a thermal joint with the same properties.

The sintering process was carried out in a Binder FD Classic Line (Binder GmbH, Tuttlingen, Germany) convection dryer at 230 °C for 1 h preceded by a 1 h heating period with a 4 °C/min temperature increase. Once sintering was complete, the samples were slowly cooled to avoid thermal shock, which could weaken their mechanical properties or lead to damage. In addition, a separate sintering experiment was carried out at a temperature of 300 °C (2:3 HC batch material).

### 2.2. Methods

**Figure 2:**
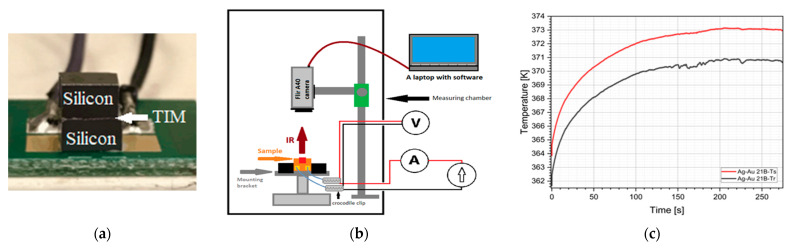
Thermographic measurements: (**a**) prepared sample; (**b**) measurement stand; and (**c**) temperatures on source and receiver surfaces.

**Thermal Analysis.** The specimens for thermographic analysis were made of thick silicon (3000 μm) to allow for precise alignment of the measurement areas on both surfaces and within the TIM region, where the geometry was adapted to the used equipment requirements. They were attached to FR-4 wafers using Amepox’s silver-based conductive adhesive, applied using a 400 μm thick polyimide film template. The silicon chip was then mounted using the MM500 (Mechatronika, Warsaw, Poland) pick-and-place robotic system. The adhesive was cured in a JEM 310 (OK Industries, Mumbai, India) convection oven for 15–20 min at 150 °C, after which the samples were cleaned with isopropanol. The final step was to solder the power wires and apply a 0.98 emissivity layer using Helling 3-D Laserscanning Anti-Glare-Spray (Helling GmbH, Heidgraben, Germany) (Figure 2a), where the delivered electrical power was controlled by a power supply and monitored by multimeters for voltage and current measurements. The samples were placed in a measurement chamber (Figure 2b) with monitored environmental conditions. Measurements were calibrated taking into account the atmospheric temperature and humidity of the air in the measurement chamber, the distance between the camera and analyzed object, and the reflected (apparent) temperature, that is, the mean temperature of the surrounding objects. The heat source was powered to maintain steady-state conditions, ensuring a stable temperature of 100 °C or 373 K (Figure 2c). The temperature difference between the heat source and receiver was recorded, and the thermal resistance (R_TH_) was calculated using Formula (1):

(1) $$R_{TH}=\frac{\Delta T}{P}=\frac{T_{S}\;-\;T_{R}}{U\;\cdot \;I}\;\left[\frac{K}{W}\right]$$

where T_S_—source temperature [K]; T_R_—receiver temperature [K]; U—source voltage [V]; I—source current [A]. The lower the thermal resistance, the better, as it allows for more efficient heat flow through the thermal joint. Temperatures were measured using a Flir A40m infrared camera (Teledyne FLIR, Wilsonville, OR, USA).

**Figure 3:**
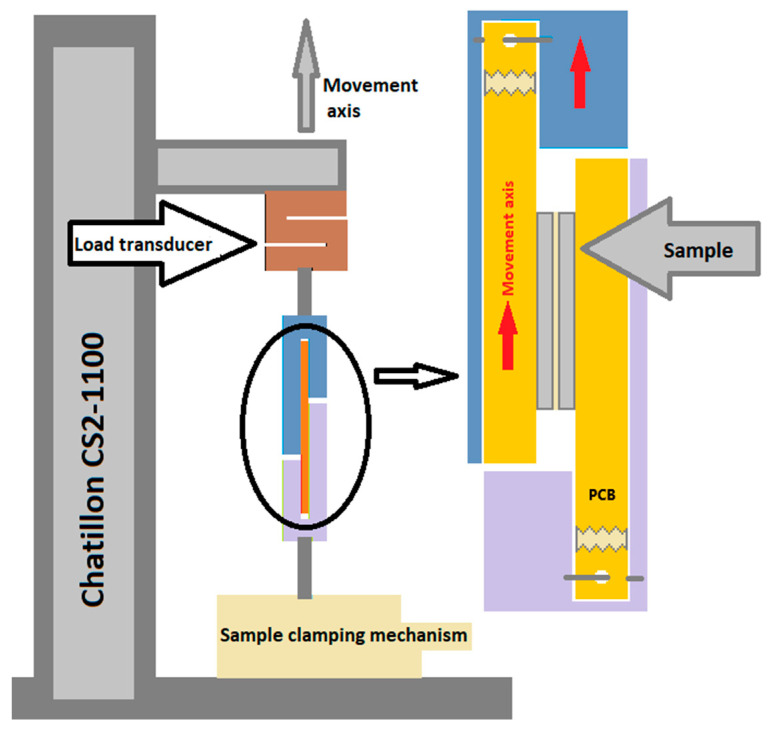
Shear strength testing station.

**Mechanical analysis** was conducted to evaluate the shear force of the sintered materials on a Chatillon CS2-1100 (Ametek, American Machine and Metals, Berwyn, PA, USA) stretching machine. The CS2-1100 ensures automatic data acquisition up to 1000 Hz with several different test stop conditions (force drop, sample break, max force/elongation, cycles, etc.). The samples for these tests were prepared using 400 µm thick silicon substrates, which were glued onto FR-4 laminates using cyanoacrylate adhesive. The prepared samples were placed in 3D-printed grips. A micrometer screw was used to precisely position the sample so that it was perpendicular to the substrate. The grips used ensured stable, vertical positioning of the sample during measurements and caused the force to act along the plane of the sample surface. Samples were tested in the pull-to-break regime, and the load cell limit was 1000 lbs (~4500 N). The higher the shear strength, the better. A schematic view of the experimental setup is shown in Figure 3.

**The thickness and homogeneity** of the thermal interface layer between the chips was assessed using a destructive method, specifically metallographic specimen analysis. Random samples (two from each batch) were taken, placed in molds, and flooded with SpeciFix Resin (Struers, Ballerup, Denmark), an epoxy resin used at a ratio of 2.5:1 with SpeciFix Curing Agent (Struers). After the removal of air bubbles and curing for about 24 h, the specimens were sanded, starting with 80-grit sandpaper and finishing with 2000-grit, and then polished with diamond micropowder polish (700 nm). The finished sections were mounted on slides, allowing them to be observed under an optical microscope.

**Optical microscopy.** For the purposes of optical inspection of the sintered TIM layers, metallographic cross-sections were prepared, exposing the cross-section of selected samples, where images were obtained by a ZEISS Axiooptical microscope (Carl Zeiss AG, Oberkochen, Germany). X-ray imaging was performed by an X-ray microtomograph (micro-CT) made by GE Nanomex|180, General Electric (GE), Berlin, Germany. Image thresholding was performed with the use of custom-made software.

The gold nanoparticle microstructure was investigated using high-resolution transmission electron microscopy (HRTEM) with an FEI Tecnai G2 20 XTWIN instrument (Hillsboro, OR, USA). A thermogravimetric study of the gold colloid after freeze-drying was carried out using a TGA2 thermowell (Metter Toledo, Greifensee, Switzerland). The measurement was carried out over a temperature range of 25–600 °C at a heating rate of 10 °C/min in an air atmosphere, using a corundum crucible with a capacity of 70 μL.

## 3. Results

### 3.1. Gold and Silver Morphology

**Figure 4:**
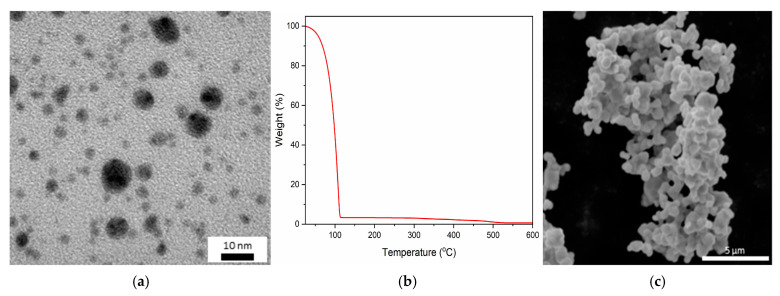
AuNPs: (**a**) TEM images; (**b**) TGA analysis of colloids after a lyophilization process; (**c**) SEM images of silver microplates.

The obtained gold nanoparticles exhibited a spherical shape and a size ranging from 2 to 10 nm (Figure 4a). Thermogravimetric (TG) analysis of the colloid after lyophilization revealed that the main mass loss (96.77%) occurred at 108 °C, corresponding to the removal of water from the solution. A second, significantly smaller mass loss (2.53%) was observed at 493 °C and was associated with the decomposition of the stabilizing polymer—polyvinylpyrrolidone (PVP). The final nanoparticle colloid contained gold at a concentration of 0.69 wt% (Figure 4b). Since the removal of PVP occurs only at temperatures close to 500 °C, and the TIM layers were fabricated within the temperature range of 200–300 °C, it should be noted that the gold nanoparticles present in these layers remained coated with the stabilizing polymer layer. The silver microparticles used to obtain the TIM structure had a spherical shape and a size of about 1 μm (Figure 4c).

### 3.2. TIM Layer Morphology

**Figure 5:**
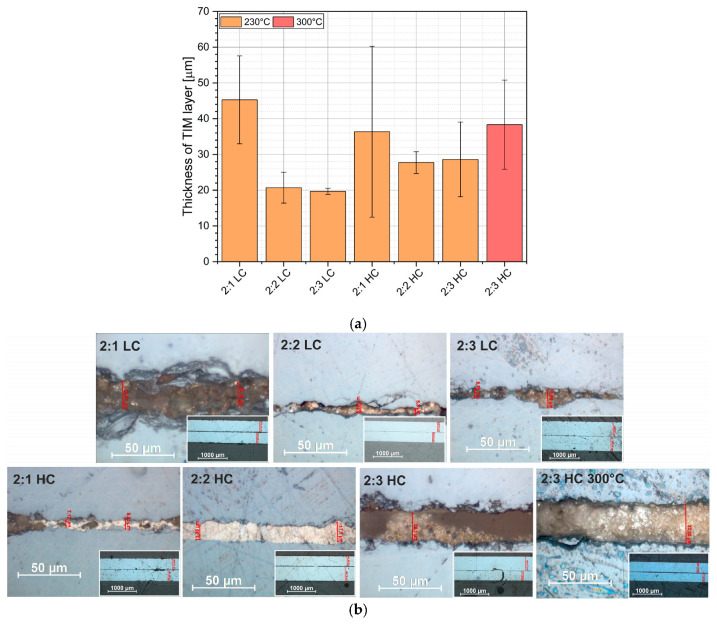

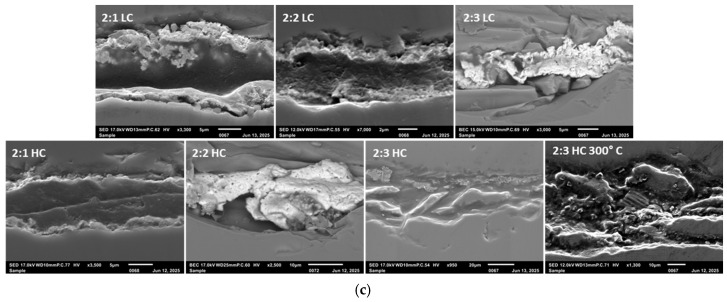
Measurements of metallographic micro sections: (**a**) optical microscope image of sample, (**b**) SEM image, and (**c**) average thickness of sintered layers.

For the purposes of optical inspection of the sintered TIM layers, metallographic cross-sections were prepared, exposing the cross-sections of select samples. In analyzing the microscopic images (Figure 5a), discontinuities in the TIM layer can be observed. This may have been caused by the grinding process, which “detaches” weakly bonded particles. The presence of loose silver–gold particles may result from the formation of pores in the sintered layer structure and insufficient bonding of these particles.

Based on the microscopic images, the thickness of the TIM layer was measured for all materials containing gold nanoparticles by calculating the arithmetic mean of the thickness values obtained at three points for each sample (Figure 5b). The results are presented in the graph. As observed, materials with a higher content of gold nanoparticles and a greater amount of solvent exhibited a thinner TIM layer (2:2 LC, 2:3 LC). However, it should be noted that the measurement of the sintered layer thickness did not clearly indicate the influence of nanoparticles on the sinter thickness due to the destructive nature of the measurement method. Optical thickness measurements primarily reflect the distribution of larger fractions of the sintered material, which may not necessarily correlate with the results of shear strength analysis or thermal resistance measurements. The analysis of cross-sections performed using SEM allowed for detailed observation of the layer’s microstructure. The images clearly show the metallic silver layer deposited on the silicon substrates, as well as the TIM layer located between them. For SEM analysis of the interface structure, we selected a continuous and poreless area (Figure 5c).

**Figure 6:**
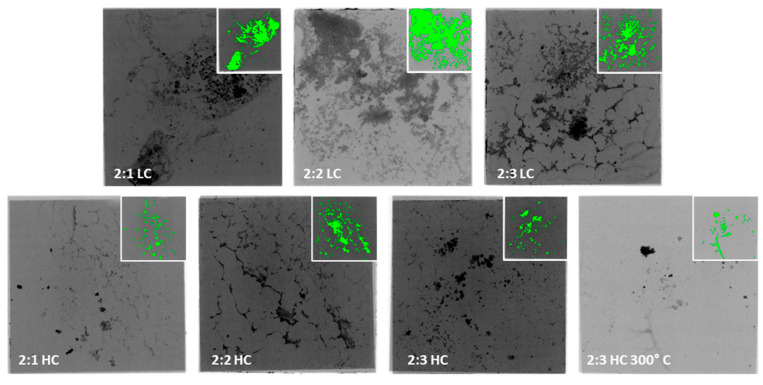
X-ray images of the TIM material series tested, showing an example of image thresholding.

**Figure 7:**
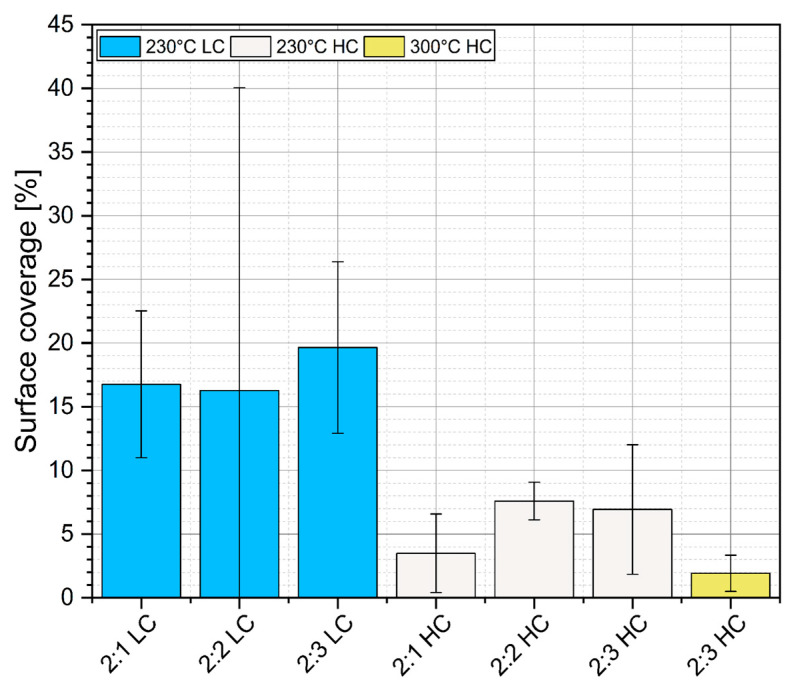
Surface coverage of TIM’s layer (background colors correspond to a particular set of samples).

In order to assess the quality and coverage of silicon chips with the TIM layer, a series of X-ray images of the sintered samples was taken (Figure 6). Then, using advanced image thresholding software, the images were analyzed to identify voids and defects, and to examine the quality of the sintered joints, with the coverage ratio calculated through image processing (Figure 7). In analyzing the results of the measurements, it can be concluded that materials containing twice as much solvent (2:1 LC, 2:2 LC, 2:3 LC) were characterized by the highest surface coverage of the chips, while reducing the solvent content drastically decreased the quality of the surface coverage. This is related to the lower viscosity of the paste, allowing for a more even coverage of the substrate to be obtained. Due to the use of gold nanoparticles in the materials, which cannot be visualized in X-ray images, there is a possibility that these surfaces were covered with the tested material so uniformly that it was not accurately detected. This method can effectively identify only agglomerates of sintered particles and pores present on the chip contact surface [23].

### 3.3. Thermal Analysis

**Figure 8:**
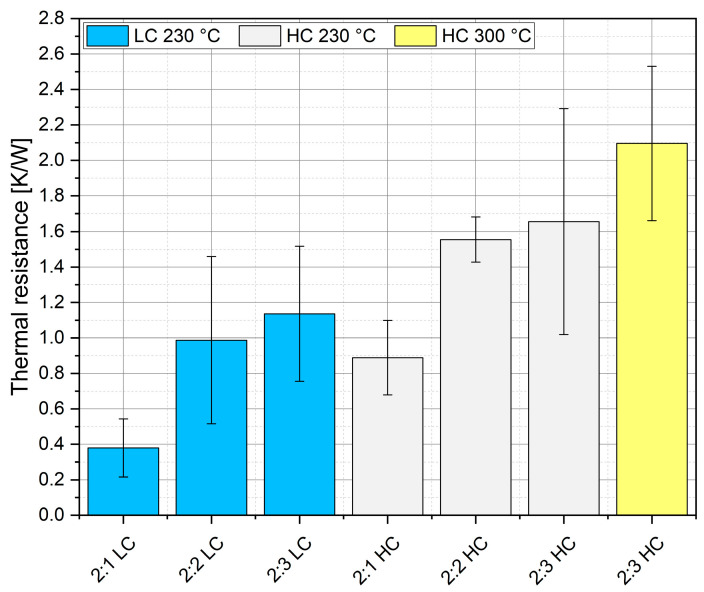
Thermal resistance chart (background colors correspond to a particular set of samples).

Figure 8 shows the results of the thermal resistance measurements for layers containing gold nanoparticles. As can be observed, the thermal resistance values obtained in the measurements of sintered layers were significantly higher than the thermal resistance values for the bulk layers, regardless of whether they contained silver or gold. This conclusion is expected, given the characteristic feature of sintered joints, the surface of which will never perfectly reproduce a continuous layer, resulting in roughness of either the silver or gold (Figure 7). Thermal resistance was calculated according to Formula (1), where power, temperature difference, and thermal resistance determined accordingly (Table 3).

Another conclusion arising directly from the analysis of the results is that the difference in thermal resistance across the series (between the lowest resistance value of 2:2 LC and the highest of 2:3 HC at 300 °C), is equal to 70%, indicating the significant influence of solvents and process temperature on the resistance value. It can be seen that the influence of gold increased the thermal resistance and directly correlated with the thermal conductivity of gold (continuous layer). The thermal resistance of gold is significantly higher than the thermal resistance of silver, so it is understandable that the addition of gold will increase both the process temperature and the thermal resistance (Figure 8).

### 3.4. Mechanical Analysis

**Figure 9:**
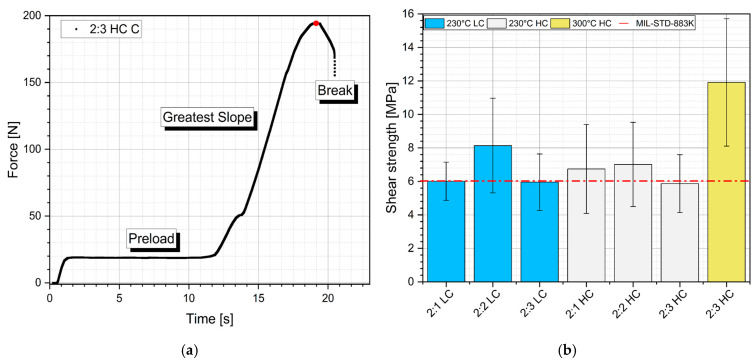
Example of shear measurement (**a**), and shear strength graph (**b**) sample 2:3 HC C (background colors correspond to a particular set of samples).

In the process of measuring the force at which the specimens broke, a graph was obtained for each specimen, indicating the value of force at which the specimen failed (Figure 9a). We analyzed maximal shear strength, that was marked with red dot in the Figure 9a. In order to present the measurement results in the form of shear strength, it was necessary to convert the value of the obtained force into strength, calculated using Formula (2):

(2) $$\tau =\frac{F}{A}\;[MPa]$$

where *τ*—sheer strength [MPa]; *F*—load force [N]; *A*—sample area [m^2^]. For these calculations, the sample area was considered as a theoretical 5 × 5 mm^2^. Shear measurements for materials containing gold nanoparticles showed that almost all of the materials tested were within the American standard describing shear strength for 5 × 5 mm^2^ chips [24]. It can be observed that the 2:3 LC and 2:3 HC materials did not meet these requirements, as their strength was slightly lower than the 6 MPa described in the standard. It should be noted that the Ag-Au 3 material, which contained a higher amount of nano-gold in its composition relative to the Ag-Au 1 material, had a higher strength with the same solvent content in the composition. This may indicate an improvement in mechanical properties with an increase in the content of gold nanoparticles (Figure 9).

Particularly noteworthy is the 2:3 HC material sintered at 300 °C. It achieved the highest mechanical strength of close to 12 MPa, which indicates a significant increase in shear strength as the temperature of the process increased. The increase in temperature is beneficial in the sintering of materials containing gold nanoparticles due to the higher melting point of gold relative to silver.

### 3.5. Comparative Analysis

**Figure 10:**
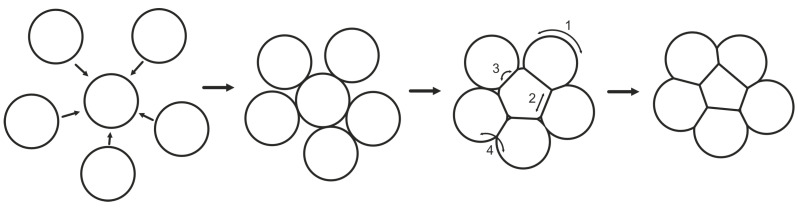
Mechanisms of mass transport: (1) surface diffusion; (2) diffusion across grain boundaries; (3) volumetric diffusion with vacancies at grain boundaries; (4) volumetric diffusion with vacancies at convex surfaces of particles. Illustration of sintering stages with special emphasis on changes in pore structure during sintering.

Considering the many factors affecting the quality of the material compositions studied in this article, one cannot overlook the essence of the sintering process. The essence of the sintering process is increases in the contribution of surface energy generated by the thin layer [17], which in turn reduces the diffusion distance at the grain boundary [25,26], thus facilitating diffusion, aggregation, and particle accumulation (neck formation) [17]. The size effect allows for a significant reduction of the sintering temperature compared to the typical melting temperature of the bulk material [27,28]. Sintering of solid phases gradually leads to a decrease in free enthalpy and a reduction in the distance between particles [29]. Mechanisms such as surface and bulk diffusion, evaporation, or condensation, as well as grain–boundary diffusion, lead to the formation of a neck between the sintered particles [28,29,30]. The stages of the sintering process and neck formation and sintering stages are shown in Figure 10 [31,32].

**Figure 11:**
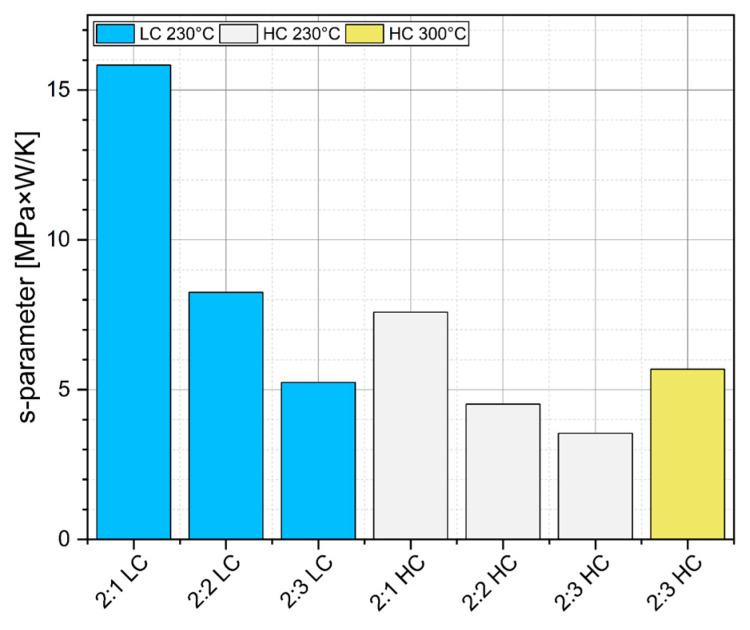
Results of “s” parameter calculations (background colors correspond to a particular set of samples).

As a result of these processes, the sintered layer becomes homogeneous (with minimal porosity) [30] and, due to the increased packing density of the particles, there is an improvement in the mechanical strength of the bond and a significant increase in thermal conductivity [29,32]. With this in mind, a comparative parameter was introduced that took into account the two most critical properties (with respect to sintered-layer quality): shear strength and thermal resistance. The ‘s’ parameter, defined by Equation (3), is their ratio; the higher its value, the better. The results of the comparative analysis are presented in Table 4 and the chart in Figure 11.

(3) $$s=\frac{\tau }{R_{TH}}=\frac{\tau \cdot P}{\Delta T}\left[\frac{MPa\cdot W}{K}\right]$$

As shown in the results in Table 4 and the chart in Figure 11, the highest value of the parameter ‘s’ was achieved by the 2:1 LC material. This stems directly from the previously obtained tensile strength data, where this material reached a relatively high strength of 8 MPa, while at the same time exhibiting low thermal resistance. It is worth noting that there is a correlation between the ‘s’ parameter and other analytical results, such as the TIM layer thickness and layer morphology assessment. As expected, the proper ratio of TIM components yields not only good mechanical strength and low thermal resistance but also a uniform layer (evenly distributed over the silicon chip surface) with an optimal thickness of approximately 20 µm. Moreover, when reviewing the comparative results, one can see that although the 2:3 HC material sintered at elevated temperature demonstrated excellent mechanical, electrical, and structural properties, it also had the highest thermal resistance among the series tested, indicating that it should be excluded from further experimental studies.

A comparison of thermal resistance of different types of TIMs is presented in Table 5. The last one entry in the table, presented in this paper, show comparable thermal resistance results to those of other authors. It is worth mentioning that among the listed papers in Table 5, TIM materials have mainly been investigated for their thermal properties, neglecting mechanical strength.

## 4. Conclusions

This study analyzed six different compositions of thermally conductive materials containing gold nanoparticles and silver microparticles. Mechanical strength analyses showed that the shear strength was significantly affected by both the temperature of the sintering process and the proportion of the mixture components. This is consistent with the physics of the sintering process [27]. The analyses showed that almost all of the materials tested meet the US military standards. Analysis of both the thermal resistance and thickness of the sintered layer showed that these parameters correlate with both the ratio of gold to silver microparticles and the solvent content. Materials containing more solvent had noticeably better homogeneity, resulting in a thinner sintered layer and better heat and electricity conductivity. The comparative analysis clearly identified several potentially commercially useful materials, while also showing that the best of the materials tested was 2:1 LC.

All samples had high and similar mechanical strength, so the crucial factor was thermal resistance. Analysis of the threshold coverage in the X-ray images showed no relation to mechanical strength measurements. Materials marked with the symbol LC had a significantly higher TIM coverage coefficient than those marked HC, while mechanical strength was at a similar level. In our opinion, high mechanical strength is the result of silver plating of the silicon dies during sample preparation. The performed analyses showed several areas suitable for further extensive study. Our future research will be focused on the optimization of TIM material composition, mostly by changing the ratio of the particles, the amount of applied solvents, and other formulation parameters. Additionally, we are going to modify the deposition process, giving extra consideration to variables such as droplet size, dispensing speed, and environmental conditions, to improve the repeatability and consistency of the material application. In order to analyze the performance and durability of developed materials in real applications, we are going to evaluate their long-term stability and reliability under extreme conditions, such as damp heat tests and temperature cycling. Our work will contribute to the development of more reliable, effective, and scalable solutions for practical use in electronics packaging.

## Figures and Tables

**Table 1:** Magnetron sputtering process conditions.

Material	I [A]	P [W]	U [V]	t [min]	FL [sccm]	p [mbar]
Ag	0.3	290	960	4	5	3.2 × 10^−3^

**Table 2:** Composition of the investigated Au-Ag TIM mixtures (background colors correspond to a particular set of samples).

Sample Composition	Metal Particles		Solvent		Notes
	Silver Microparticles	Gold Nanoparticles	Polyethylene Glycol	Ethyl Alcohol	
Sample Description	Weight Ratio				
2:1 LC	2	0.01	7	5	LC—low concentration of metal particles
2:2 LC	2	0.02			
2:3 LC	2	0.03			
2:1 HC	2	0.01	3	2	HC—high concentration of metal particles
2:2 HC	2	0.02			
2:3 HC	2	0.03			

**Table 3:** Electrical (voltage, current, and power) and thermal (source, receiver temperatures, and their difference) analysis and thermal resistance calculations (background colors correspond to a particular set of samples).

Sample No.	Voltage	Current	Power	Source Temperature	Receiver Temperature	Temperature Difference	Thermal Resistance
	U [V]	I [A]	P [W]	T_S_ [K]	T_R_ [K]	ΔT [K]	$R_{\mathrm{TH}}\;\left[\frac{K}{W}\right]$
2:1 LC	0.33	6.35	2.11	372.50	371.70	0.80	0.38
2:2 LC	0.32	6.28	2.02	369.00	367.00	2.00	0.99
2:3 LC	0.36	6.13	2.20	372.20	369.70	2.50	1.14
2:1 HC	0.42	5.31	2.25	371.80	369.80	2.00	0.89
2:2 HC	0.20	6.48	1.29	373.20	371.20	2.00	1.55
2:3 HC	0.19	6.47	1.21	373.30	371.30	2.00	1.65
2:3 HC	0.33	4.37	1.43	372.80	369.80	3.00	2.09

**Table 4:** Results of measuring the “s” parameter (background colors correspond to a particular set of samples).

	Shear Strength [MPa]	Thermal Resistance [$\frac{K}{W}$]	‘s’ Parameter [$\frac{MPa\cdot W}{K}$]
2:1 LC	6.01	0.38	15.83
2:2 LC	8.14	0.99	8.24
2:3 LC	5.95	1.14	5.24
2:1 HC	6.74	0.89	7.59
2:2 HC	7.01	1.55	4.51
2:3 HC	5.87	1.65	3.54
2:3 HC_300_	11.9	2.09	5.69

**Table 5:** Comparison of thermal resistance of different types of thermal interface materials.

TIM Type	Thermal Resistance	References
Adhesives	0.256 K/W (16.4 mm × 16.4 mm), 69 mm^2^ K/W	[33,34]
Gels	0.134 K/W (16.4 mm × 16.4 mm), 36 mm^2^ K/W	[34]
Thermal grease	0.121–0.252 K/W	[35]
Gap pad	0.480–0.668 K/W	[35]
Phase change materials	0.192–0.209 K/W	[35]
Epoxy based micro- and nano-silver TIM	1.3–10.26 K/W	[4]
2:1 LC	0.38 K/W	Presented paper

## Data Availability

The original contributions presented in the study are included in the article, further inquiries can be directed to the corresponding author.

## Abbreviations

The following abbreviations are used in this manuscript:

TIM	Thermal interface material
MIL-STD	Military standard
LC	Low concentration
HC	High concentration
PVP	Polyvinylpyrrolidone
FR-4	Flame Retardant 4
HRTEM	High-resolution transmission electron microscopy
TGA	Thermogravimetry
SEM	Scanning electron microscopy
